# Tumor Mesenchymal Stromal Cells Regulate Cell Migration of Atypical Teratoid Rhabdoid Tumor through Exosome-Mediated miR155/SMARCA4 Pathway

**DOI:** 10.3390/cancers11050720

**Published:** 2019-05-24

**Authors:** Yi-Ping Yang, Phan Nguyen Nhi Nguyen, Hsin-I Ma, Wen-Jin Ho, Yi-Wei Chen, Yueh Chien, Aliaksandr A. Yarmishyn, Pin-I Huang, Wen-Liang Lo, Chien-Ying Wang, Yung-Yang Liu, Yi-Yen Lee, Chien-Min Lin, Ming-Teh Chen, Mong-Lien Wang

**Affiliations:** 1 Department of Medical Research, Taipei Veterans General Hospital, Taipei 112, Taiwan; molly0103@gmail.com (Y.-P.Y.); g39005005@gmail.com (Y.C.); yarmishyn@gmail.com (A.A.Y.); 2 School of Pharmaceutical Science, National Yang Ming University, Taipei 112, Taiwan; 3 School of Medicine, National Yang-Ming University, Taipei 112, Taiwan; nguyennhi204@hotmail.com (P.N.N.N.); keyw4928067@gmail.com (W.-J.H.); chenyw@vghtpe.gov.tw (Y.-W.C.); pihuang@vghtpe.gov.tw (P.-I.H.); yylee62@gmail.com (Y.-Y.L.); mtchen@vghtpe.gov.tw (M.-T.C.); 4 Cancer Center, Taipei Veterans General Hospital, Taipei 112, Taiwan; 5 Graduate Institute of Medical Sciences, National Defense Medical Center, Taipei 114, Taiwan; uf004693@mail2000.com.tw; 6 Department of Neurological Surgery, Tri-Service General Hospital and National Defense Medical Center, Taipei 114, Taiwan; 7 Division of Oral and Maxillofacial Surgery, Taipei Veterans General Hospital, Taipei 112, Taiwan; wllo@vghtpe.gov.tw; 8 Department of Dentistry and Institute of Oral Biology, National Yang-Ming University, Taipei 112, Taiwan; 9 Institute of Clinical Medicine, National Yang-Ming University, Taipei 112, Taiwan; wangcy@vghtpe.gov.tw (C.-Y.W.); yyliu@vghtpe.gov.tw (Y.-Y.L.); 10 Department of Surgery, Taipei Veterans General Hospital, Taipei 112, Taiwan; 11 Chest Department, Taipei Veterans General Hospital, Taipei 112, Taiwan; 12 Department of Neurosurgery, Neurological Institute, Taipei Veterans General Hospital & National Yang-Ming University, Taipei 112, Taiwan; 13 Department of Neurosurgery, Shuang-Ho Hospital, New Taipei City 235, Taiwan; m513092004@tmu.edu.tw; 14 Taipei Neuroscience Institute, Taipei Medical University, Taipei City 110, Taiwan; 15 Institute of Food Safety and Health Risk Assessment, National Yang Ming University, Taipei 112, Taiwan

**Keywords:** atypical teratoid/rhabdoid tumor, tumor-associated mesenchymal stem cells, miR155, SMARCA4, exosome

## Abstract

Atypical teratoid/rhabdoid tumor (ATRT) is a rare pediatric brain tumor with extremely high aggressiveness and poor prognosis. The tumor microenvironment is regulated by a complex interaction among distinct cell types, yet the crosstalk between tumor-associated mesenchymal stem cells (tMSCs) and naïve ATRT cells are unclear. In this study, we sought to identify the secretory factor(s) that is responsible for the tMSC-mediated regulation of ATRT migration. Comparing with ATRT cell alone, co-culture of tMSCs or addition of its conditioned medium (tMSC-CM) promoted the migration of ATRT, and this effect could be abrogated by exosome release inhibitor GW4869. The exosomes in tMSC-CM were detected by transmission electron microscope and flow cytometry. ATRT naïve cell-derived conditioned media (ATRT-CM) also enhanced the exosome secretion from tMSCs, indicating the interplay between ATRT cells and tMSCs. Microarray analysis revealed that, compared with that in bone marrow-derived MSCs, microRNA155 is the most upregulated microRNA in the tMSC-CM. Tracing the PK67-labeled exosomes secreted from tMSCs confirmed their incorporation into naïve ATRT cells. After entering ATRT cells, miR155 promoted ATRT cell migration by directly targeting *SMARCA4*. Knockdown of *SMARCA4* mimicked the miR155-driven ATRT cell migration, whereas *SMARCA4* overexpression or the delivery of exosomes with miR155 knockdown suppressed the migration. Furthermore, abrogation of exosome release with GW4869 reduced the tumorigenesis of the xenograft containing naïve ATRT cells and tMSCs in immunocompromised recipients. In conclusion, our data have demonstrated that tMSCs secreted miR155-enriched exosomes, and the exosome incorporation and miR155 delivery further promoted migration in ATRT cells via a *SMARCA4*-dependent mechanism.

## 1. Introduction

An extremely aggressive and highly malignant embryonal central nervous system tumor, atypical teratoid rhabdoid tumor (ATRT), accounts for 1%–2% of all pediatric tumors [1]. ATRT is a rare tumor that predominantly affects children younger than three years old, with only a few cases in adults [2,3], and usually carries extremely poor overall survival [2]. Due to the presence of a combination of rhabdoid cells and neuroectodermal, epithelial and mesenchymal elements, ATRT can be distinguished from primitive neuroectodermal tumor or medulloblastoma regardless of their histologic similarities [4]. Despite the advanced cancer treatment, the standard therapy for ATRT is usually ineffective. Craniospinal irradiation is advised to avoid in younger children <3 years of age due to long-term neurocognitive and neuroendocrine consequences. High-dose chemotherapy may be an alternative approach to radiation therapy. However, this approach has a poor outcome with relatively high rates of recurrence and usually requires repeated surgery and chemotherapy. Recent studies have shown that tumor progression is the major leading cause of mortality in ATRT patients [5]. Since ATRT is a rare disease, much remains unknown about the molecular mechanisms governing tumor growth and development. Hence, understanding the mechanisms regulating tumorigenesis in ATRT is crucial.

Cancer stem cells (CSCs) is a small subset of cells within tumors that is thought to have the highest tumorigenic capabilities and is responsible for tumor relapse [6]. Increasing evidence support the notion that regulation of survival and behavior of CSCs is modulated by surrounding microenvironment factors, such as mesenchymal stem cells (MSCs), tumor-associated mesenchymal/stromal cells (tMSCs), endothelial cells, cytokines and growth factors [7,8]. Among cells of the microenvironment, mesenchymal stem cell and tMSCs have recently generated a major interest as they are recruited into microenvironment and favor the tumor progression and metastasis [9]. Mesenchymal stem cells (MSC), found in various areas including the bone marrow and adipose tissue, interact with CSCs to manipulate their environment and support cancer progression through paracrine signaling. Interestingly, the crosstalk between CSCs, MSCs, tMSCs, and tumor microenvironment is complex and can be direct, involving Notch signaling, gap junctional intercellular communication and nanotube formation, or indirect, involving the transfer of genetic material by the shuttling of exosomes, the exchange of cytokines/chemokines [10]. Due to the complexity of ATRT, the roles of tMSC-like cells residing in ATRT and regulating the CSC-like properties o are not yet determined. Furthermore, despite the fact that exosome-driven paracrine signaling is a subject of research, the tMSCs-ATRT crosstalk mediated by secreted exosomes is still poorly understood. Therefore, the interplay between tMSCs and ATRT through secretion of exosomes is the main focus of this study.

Exosomes are 50 nm to 150 nm extracellular vesicles released from cells [11]. Due to the origin from multi-vesicular endosomes, exosomes are enriched in the tetraspanin family of proteins (CD63, CD9 and CD81). Evidence emerged that exosome-mediated transfer of different cargos containing a variety of bio-macromolecules, including nucleic acids and proteins support cancer progression and spreading [12]. Tumor cells secrete exosomes to enhance tumorigenicity through interacting with cells in the microenvironment. For instance, glioma cells secrete exosomes to modulate their surroundings to tumor-promoting microenvironment through the transfer of the oncogenic receptor epidermal growth factor receptor variant III (EGFRvIII) [13]. Alternatively, exosomes secreted from mesenchymal cells are transferred to tumor cells and thus facilitate tumor development. Exosome-mediated tumor-stromal communication causes larger tumor burden and reduced host survival in orthotopic xenografts [14]. Exosome-carrying microRNAs (miRNAs) have recently been determined as the main candidates of paracrine interaction between MSCs and tumors that are responsible for effects on the phenotypic changes of tumor cells.

MicroRNAs (miRNAs) are small non-coding RNAs that post-transcriptionally regulate gene repression [15]. miRNAs can be oncogenic and involved in many aspects of tumor progression. The expression levels of biomarker miRNAs in cerebellar neuronal progenitors and tumors, such as miR-125b, miR-326, and miR-324-5p can be used to predict prognosis and patient outcome [16]. Upregulation of miR-155 is associated with breast cancer progression [17] and can be used as a diagnostic biomarker for cancer detection [18]. Tumor environment can modulate the carcinomatous malignancy and metastasis in refractory and recurrent ATRTs through the paracrine effect of miR-155. Therefore, in this study, we isolated tMSCs and stably cultivated them in vitro. We demonstrated that tMSCs, which are the major type of mesenchymal stromal cells, play a pivotal role in ATRT tumor cell migration, through exosome-delivered miR-155. Neutralizing exosomes and exosomal miR-155 in the tMSC-conditioned medium hindered its enhanced effect on ATRT malignancy. The present study identified exosomal miR-155 as a crucial factor that paracrinally mediates the tMSC-dependent promotion of ATRT malignancy through direct targeting of *SMARCA4*.

## 2. Materials and Methods

### 2.1. Culture and Maintenance of ATRT Cells and tMSCs

The isolation and culture of ATRT cells and tMSCs were conducted by following the studies reported by Liu et al. [19] and Zhang et al. [20], respectively. All procedures for the sample acquisition followed the tenets of the Declaration of Helsinki and have been approved by the Institutional Review Committee at Taipei Veterans General Hospital (Approval No. 2012-12-021B). From January 1998 to April 2011, a total of thirty-two patients who were diagnosed as ATRT by neurology physicians have received treatment in Taipei Veterans General Hospital [21,22,23]. After the surgical removal of ATRT tissues, the tissues were washed three times using the Hanks’ balanced salt solution (HBSS; Invitrogen/Life Technologies, Carlsbad, CA, USA) supplemented with glucose. Subsequently, washed ATRT tissues were cut into slices at a 300 mm thickness and immersed in glucose-supplemented HBSS containing 0.1% (w/w) collagenase (Sevapharma, Prague, Czech Republic) at 37 °C for 15 min on a rotation shaker at 125 rpm. After the collagenase dissociation, all cells were then incubated in RPMI 1640 medium (Gibco®, Carlsbad, CA, USA) supplemented with 10% fetal bovine serum (FBS) and antibiotics, and cultivated in 5% CO_2_ atmosphere under 37 °C incubation. Characteristics of the cells were reported in our previous studies [21,22,24,25] and were negative for integrase interactor 1 (INI1) and positive for the transcription activator BRG1. Cell morphology was analyzed using the Zeiss Axiovert 25 Phase Contrast Inverted Microscope (Zeiss, Oberkochen, Germany), and the digital images were captured using a Canon Power Shot G10 equipped with a Carl Zeiss 426,126 lens. For the isolation of tMSCs, the ATRT tissue was cut into 1 mm^3^ and incubated with L-DMEM containing 10% FBS, 100 U/mL penicillin and 100 U/mL streptomycin in 5% CO_2_ atmosphere under 37 °C temperature. The medium was changed every three days. Generally, fibroblast-like cells could be observed approximately 10 days after the initial plating. Subsequently, the cells were trypsinized and passed into a new flask for expansion. The isolated tMSCs were characterized by flow cytometry analysis to assess the expression of surface markers (positive for MSC-specific markers—CD29, CD44, CD90, CD105, and CD166—and negative for the endothelial and hematopoietic cell markers—CD31, CD34, and CD45; Supplementary Figure S1). Cells at passage three and four were used in all experiments in this study. The experiments were undertaken with the understanding and written consent of each subject.

### 2.2. Mir-155 Sponge Construction

MiR-155 SPONGE, scramble and its anti-sense microRNAs were constructed using a pcDNA™6.2-GW/EmGFP-miR vector (Invitrogen, Carlsbad, CA, USA) following the study by Chiou et al [26]. MicroRNA SPONGE sequence design was based on a previous report [27]. Further amplifications were conducted with the recovery of BamH1- and XhoI-digested fragments and were subsequently subcloned into the pcDNA 6.2-GW/EmGFP-miR plasmid. After the construction, all expression vectors were sequenced and verified. The transfected ATRT cells were cultured and maintained in the culture medium containing blasticidin. The pcDNA 6.2-GW/EmGFP-miR-neg control plasmid contains an insert that can form a hairpin structure that is processed into mature miRNA but is predicted not to target any known vertebrate gene.

### 2.3. Exosome Purification from Conditioned Medium and Identification by CD63 Surface Marker

Cells and the suspension were centrifuged at 300 × g for 10 min at 4 °C to remove the cell pellet and large particles or debris. The supernatant was further centrifuged at 16,500 × g for 20 min at 4 °C. The pellets were discarded and the supernatant was collected and filtered by 20 nm filter. The filtered supernatant was ultracentrifuged (Beckman Coulter Optima L-100K, SW32 Ti rotor, USA) at 100,000 × g, 90 min at 4 °C. The supernatant was discarded and the exosome pellets were re-suspended in 10–30 µL PBS. Purified exosomes were stored at −80 °C for future study. For evaluating the expression of CD63 exosome marker on our purified exosomes, we applied Exosome-Human CD63 Isolation/Detection Reagent (Thermo Fisher Scientific, Catalog No. 10606D) to capture the exosomes with magnetic micro-beads and stained the exosome-bead complexes with anti-CD63-PE antibodies. The population of CD63-positive exosome were then evaluated by Flow Cytometry.

### 2.4. Exosome Uptake Analysis with PKH67 Labeling

PKH67 green fluorescent cell linker kit (Sigma-Aldrich) was used to label the tMSC cell membrane as well as tMSC-derived exosomes [28,29,30], according to the manufacturer’s recommendations. In brief, tMSC cells or isolated exosome from tMSC conditioned medium were placed in conical bottom polypropylene tube and wash once using serum-free medium. The suspension was centrifuged at 400 × g, room temperature; the supernatant was carefully removed. The remaining cell pellet was resuspended in 1 mL of Diluent C by gentle pipetting. Immediately prior to staining, add 4 μL of PKH67 in 1 mL Diluent C, mix well. Add the 1 mL of cell suspension or exosome to the 1 mL PKH67 solution, mix the solution immediately by pipetting, and incubate the cell/PKH67 solution for five min with periodic mixing. The labeling procedure was then stopped by adding an equal volume of serum and incubate for one minute. The labeled cells or exosome were collected by centrifugation at 400 × g for 10 min at 20–25 °C, and carefully remove the supernatant. Cells were resuspended in 10 mL of complete medium followed by another two rounds of washing with 10 mL complete medium and centrifugation to ensure the complete removal of unbound dye. After the final wash, cells or exosomes were resuspended in 10 mL complete medium for further experiments and assessments. Exosome labeling analysis was performed using the Accuri C6 Flow Cytometer System (BD Biosciences, New Jersey, USA), and exosome protein content was determined by using the Pierce™ BCA Protein Assay Kit (ThermoFisher Scientific Inc., Massachusetts, USA) before further analyses.

### 2.5. Heparin Inhibition of Exosome Uptake

For inhibiting exosome update in the recipient cells, ATRT cells were pre-treated with 10 μg/mL heparin for four hours before incubation with 20 µg purified exosome for another 24 h. ATRT cells were then collected for future analyses [31].

### 2.6. GW4869 Treatment

GW4869 was purchased from Sigma (St. Louis, MO) and dissolved in DMSO into a stock solution of 5 mM before dilution in culture supernatant to achieve 20 μM concentration in tMSC culture condition for 48 h prior to conditioned medium cultivation. After treatment, tMSCs were washed with PBS 2–3 times and cultured in their culture medium for another 24 h for collecting the conditioned medium. For in vivo xenotransplantation experiment, mice were intraperitoneally injected with GW4869 (1.25 mg/kg) on day four post-tumor implantation [28,29,30].

### 2.7. Wound Healing Assay

For wound-healing cell migration assay, 2 × 10^5^ cells were seeded into each silicon culture insert (Ibidi # 80241, Germany) in a 24-well cell culture plate and allowed to adhere overnight. Silicon inserts were removed and cells were washed with PBS twice. Each well of the 24-well plate were treated with 10 μg/mL of mitomycin C in 1 mL of culture media for 1 h, followed by PBS wash for tice and filled with 1 mL of culture medium or the mixture of DMEM culture medium with conditioned medium (1:1), and the migratory cells were imaged with an inverted microscope. Wound area recovery by migrated cells was quantified by Image J.

### 2.8. Transmission Electron Microscopy

Exosomes were visualized using transmission electron microscopy (TEM). Briefly, 30 µL of exosome suspension was fixed in 30 µL of 2% paraformaldehyde. Two microliter of this mix was transferred onto each of two Formvar-carbon coated nickel electron microscopy grids. Membranes were covered for 60 min. A 30 µL drop of PBS was placed on a sheet of parafilm and grids transferred with the sample membrane side facing down using clean forceps for two min. The grids were kept wet on the side of the membrane during all steps, but dry on the opposite side. The grids were transferred to a 30 µL drop of 1% glutaraldehyde for 10 min before transferring to a 30 µL drop of distilled water for 10 min. This was repeated seven times for a total of five water washes. To contrast the samples, grids were transferred to a 30 µL drop of uranyl-acetate solution, pH 7, for 15 min before transferring to a 30 µL drop of methyl-cellulose-UA (a mixture of 0.4% uranyl acetate and 0.13% methylcellulose in a ratio of 1:9, respectively) for 10 min, placing the grids on a glass dish covered with parafilm on ice. The grids were removed with stainless steel loops and excess fluid blotted gently on Whatman no.1 filter paper. Grids were left to dry and stored in appropriate grid storage boxes. Grids were observed with JEM 1,011 transmission electron microscope at 80 kV, at 150K magnified field.

### 2.9. Extraction of RNA in Exosomes

We used the miRNeasy Serum/Plasma Kit for exosome-RNA extraction. Add 1mL QIAzolLysis reagent to 200 μL exosome. Mix by vortexing or pipetting up and down. Place the tube containing the lysate on the benchtop at room temperature (15–25 °C) for five min. Add 200 μL chloroform to the starting sample to the tube containing the lysate and cap it securely. Vortex or shake vigorously for 15 s. Place the tube containing the lysate on the benchtop at room temperature (15–25 °C) for 2–3 min. Centrifuge for 15 min at 12,000 × g at 4 °C. Transfer the upper aqueous phase to a new collection tube. Avoid transfer of any interphase material. Add 900 μL 100% ethanol and mix thoroughly by pipetting up and down several times. Pipet up to 700 μL of the sample, including any precipitate that may have formed, into an RNeasy MinElute spin column in a 2 mL collection tube. Close the lid gently and centrifuge at ≥8000 × g (≥10,000 rpm) for 15 s at room temperature (15–25 °C). Discard the flow-through. Repeat the previous step using the remainder of the sample. Discard the flow through. Add 700 μL Buffer RWT to the RNeasy MinElute spin column. Close the lid gently and centrifuge for 15 s at ≥8000 × g (≥10,000 rpm) to wash the column. Discard the flow-through. Pipette 500 μL Buffer RPE onto the RNeasy MinElute spin column. Close the lid gently and centrifuge for 15 s at ≥8000 × g (≥10,000 rpm) to wash the column. Discard the flow-through. Pipette 500 μL of 80% ethanol onto the RNeasy MinElute spin column. Close the lid gently and centrifuge for 2 min at ≥8000 × g (≥10,000 rpm) to wash the spin column membrane. Discard the collection tube with the flow-through. Place the RNeasy MinElute spin column into a new 2 mL collection tube. Open the lid of the spin column, and centrifuge at full speed for 5 min to dry the membrane. Discard the collection tube with the flow-through. Place the RNeasy MinElute spin column in a new 1.5 mL collection tube. Add 14 μL RNase-free water directly to the center of the spin column membrane. Close the lid gently, and centrifuge for 1 min at full speed to elute the RNA.

### 2.10. Microarray Analysis for MicroRNA Expression in Exosome

Exosome-RNA extraction was using a miRNeasy kits (Qiagen, Hilden, Germany), according to the manufacturer’s instruction. RNA concentration and purity were assessed by spectrophotometric analysis. The quality of small RNAs in each sample was determined using the 2100 Bioanalyzer assay (Agilent Technologies, Santa Clara, CA, USA). MiRNA microarray experiments were performed by using the Agilent Human miRNA Microarray Kit version 3 with probe sets for 470 human miRNAs. For each sample, 100 ng total RNA was hybridized with the miRNA array and further processed according to Agilent’s miRNA Microarray System protocol. The scanned images were gridded and analyzed by using Agilent Feature Extraction Software version 10.1.

### 2.11. TaqMan® MicroRNA Assays

Total RNA was isolated using TRIzol® reagent, and the isolated RNAs were reverse-transcribed using the Superscript III first-strand synthesis system for RT–PCR (Invitrogen). For miRNAs, quantitative real-time polymerase chain reaction (qRT-PCR) was performed using TaqMan miRNA Assays (ThermoFisher Scientific) [32], according to manufacturer’s instruction, with specific primer sets (Supplementary Table S2). Briefly, for miR-155 expression measurement, we used the miR-155 primer (Applied Biosystems, Order:227746) for RT-PCR, follow by qRT-PCR using miR-155 specific forward and reverse primers (Supplementary Table S2). The small non-coding RNA RNU6B was used as an internal control. The qRT-PCR was run in the LightCycler ®480 real-time PCR system (Roche LifeScience, Indiana, USA) for a total of 45 cycles (50 °C, 2 min for hold, 95 °C, 10 min for hold, then run the 95 °C, 15 s, 60 °C, 1 min for each cycle). The qRT-PCR results were then analyzed by the SDS 7000 software (Thermo Fisher Scientific).

### 2.12. Western Blotting

Total cell lysate extraction and immunoblotting analysis were performed as described [33]. A protein sample aliquot was boiled at 95 °C for 5 min and separated on 10% SDS-PAGE. The proteins were transferred to PVDF membrane (Merck Millipore, Billerica, MA, USA). Primary and secondary antibodies were added as indicated. Reactive protein bands were detected by the enhanced chemiluminescent (ECL) detection system (Merck Millipore, Billerica, MA, USA). Used antibodies are listed in Supplemental Table S2.

### 2.13. Xenotransplantation of ATRT Cells and 3T-MRI

For the xenotransplantation of ATRT cells, all experimental procedures involving animals were conducted as previously reported [19], in accordance with the institutional animal welfare guidelines and approved the animal protocol of Taipei Veterans General Hospital. Female immunodeficient NOD-SCID mice at 7–8 weeks of age were purchased from the National Laboratory Animal Center (Taipei, Taiwan) and were intracranial (IC) injected with ATRT cells suspended in 20 mL PBS solution. For the IC injection, the skulls of SCID mice were immobilized with a stereotaxic apparatus. A 1.5-mm hole was created in the cranium with the rotating fine handheld tweezers. Following the induction of general anesthesia, aliquots of ATRT cells were injected into the frontal cortex of NOD-SCID mice. The coordinates for IC injection were 2 mm to the right of the midline, 2 mm anterior to the coronal suture and 3.5 mm deep. The tumor size during the tumorigenesis was monitored by using 3 Tesla magnetic resonance imaging (3T-MRI). Tumor volume was calculated by performing the 3T-MR imaging Biospect system (Bruker, Ettlingen, Germany) with a miniquadrature coil for radiofrequency transmission and the reception of 3T-MR imaging signals. The tumor volume was then analyzed by the Image-Pro Plus software (Media Cybernetics, Rockville, MD, USA).

### 2.14. Statistical Analysis

The results are reported as the mean ± SD. Statistical analyses were performed using Student’s t-test. A *p*-value <0.05 was considered statistically significant.

## 3. Results

### 3.1. Tumor-Associated Mesenchymal Stromal Cells Enhances Migratory Ability of ATRT Cell Lines Through an Exosome-Dependent Mechanism

**Figure 1:**
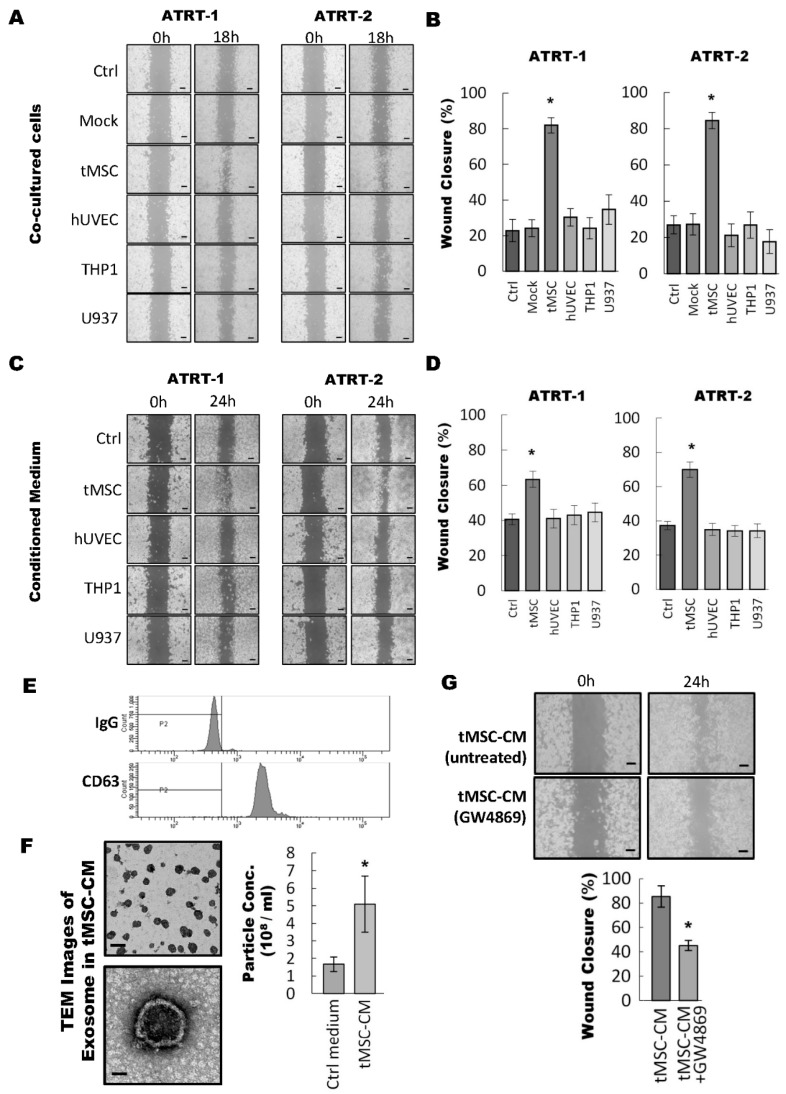
Tumor-associated mesenchymal stromal cells enhanced the migratory ability of ATRT cell lines through an exosome-dependent mechanism. (**A**,**B**) A wound healing migration assay was performed with ATRT cells co-cultured with different types of stromal cells. The ATRT-1 and ATRT-2 cell lines were seeded in the silica chamber attached in 12-well plates. The silica chambers were removed after 24 h to create the gap for cell migration, and the indicated stromal cells were seeded on the 0.2 µm filter trans-well chambers inserted in the 12-well plates for an indirect co-culture system. The cell migration was observed under a microscope for up to 18 h. Ctrl: no cells seeded in the upper chamber; Mock: the upper chambers were seeded with the same ATRT cells as the lower chamber; tMSC: tumor-associated mesenchymal stem cell; hUVEC: human umbilical vein endothelial cell, THP1: human monocytic leukemia; U937: human myeloid leukaemia (**A**). The area covered by migrated cells was calculated by Image J software and presented as a percentage in the xenografts (**B**). This experiment was done with three distinct biological replicates. * *p* < 0.05. (**C–D**) ATRT cells were subjected to a wound-healing migration assay in the presence of conditioned medium derived from different types of stromal cells. The cell migration was observed under a microscope for up to 24 h (C). The area covered by migrated cells was calculated by Image J software and presented as percentage in the grafts (D). This experiment was done with three distinct biological replicates. * *p* < 0.05. (**E**) Vesicles in tMSC conditioned medium were isolated and stained with anti-CD63 antibodies using the Exosome-Human CD63 Isolation/Detection Reagent (Thermo Fisher Scientific). The CD63-positive exosomes were analyzed by Flow Cytometry. IgG: Immunoglobulin G (**F**) Left: Exosomes in the tMSC conditioned medium were observed under transmission electron microscopy (TEM). The scale bar in the top picture represents 100 nm, while the scale bar in the bottom picture represents 50 nm. Right: control medium and tMSCs conditioned medium were subjected to quantification of vesicles/particles by nanoparticle tracking analysis (NTA). These experiments were done with three distinct biological replicates. * *p* < 0.05. (**G**) ATRT cells cultured in conditioned media derived from tMSC (tMSC-CM) treated with or without GW4869 were subjected to a wound-healing migration assay. The migrated cells were photographed at 24 h (top) and the area covered by migrated cells were calculated and presented as a percentage in the graft (bottom). This experiment was done with three distinct biological replicates. * *p* < 0.05.

The tumor microenvironment, including non-cancer cells and cells of the tumor stroma, has been recognized as a crucial factor affecting the tumor progression of various types of human cells [7,8]. To identify which cancer-associated stromal cells involved in the regulation of malignancy of ATRT, various types of stromal cells including tMSCs, human umbilical vein endothelial cells (hUVEC), THP1- and U937 monocytes were co-cultured with two different ATRT cell lines (ATRT-1 and ATRT-2) [21,22,24,25] for a wound healing migration assay (Figure 1). Compared with control (ctrl) and mock, hUVEC, THP1, and U937 failed to show consistent result in the two ATRT cell lines; while 12-h co-culturing with tMSC consistently and significantly increased the migratory capability of the two ATRT cell lines and resulted in more than 80% coverage of the created wound-healing space (Figure 1A,B). These results suggested a particularly crucial role of tMSCs in the tumor microenvironment to promote the migratory ability of ATRT cells. In addition, tMSCs were previously reported to secrete molecules in the tumor microenvironment to mediate the cell-cell interaction and the communication between cancer-associated stromal cells and tumor [9,10]. Hence, we hypothesized that enhanced cell migration of ATRT by tMSCs is modulated through secreted molecules from tMSCs. To prove the hypothesis, ATRT cells were treated with conditioned medium collected respectively from the cultures of tMSC, hUVEC, THP1, and U937 cells, followed by wound-healing migratory assays (Figure 1C,D). Exposing ATRT to the tMSCs-derived conditioned medium resulted in increased coverage of the created wound space, indicating an enhanced migratory capacity of ATRT cells (Figure 1D). Taken together, these findings indicated that tMSCs may enhance ATRT cell migration through secreted molecules in the conditioned medium.

Exosomes are a type of important extracellular microvesicle in tumor environment to regulate the interaction between tumors and their surrounding stromal cells and to enhance tumorigenicity [13,14]. Hence, we isolated and characterized vesicles obtained from tMSC-derived conditioned medium to further confirm the identity of exosomes and its essential biological role in paracrine signaling regulating ATRT tumor progression. Vesicles were isolated from tMSC-conditioned medium (tMSC-CM) and analyzed by flow cytometry using Exosome-Human CD63 Isolation/Detection Reagent (Thermo Fisher Scientific, Catalog No. 10606D) (Figure 1E). The flow cytometry results showed all isolated vesicles were stained positively with exosome surface marker—CD63. Furthermore, the diameter of the collected vesicles was examined by transmission electron microscopy (TEM) (Figure 1F). The vesicles produced from tMSCs in this study appeared to have sphere-like morphology and were found to be within the size range of 50 to 100 nm (Figure 1F, left panel). In addition, the number of vesicles obtained from tMSCs-CM was significantly (two times) higher than of those from Ctrl media as quantified by nanoparticle tracking analysis (NTA) (Figure 1F, right panel). The findings suggested the molecules produced by tMSCs were exosomes. Next, we evaluated the migratory ability of ATRT cells affected by the presence of tMSC-released exosomes. tMSCs were cultured with and without exosome inhibitor GW4869 (GW4869 and untreated, respectively) (Figure 1G). The results showed significant slowdown on the migration rate of ATRT cells when cells were cultured in the presence of exosome inhibitor GW4869 in ATRT-CM conditioned medium (Figure 1G, bottom). In summary, our data indicate that vesicles presented in tMSCs-derived conditioned medium are exosomes and that the exosomes are crucial factors in regulating the migratory ability of ATRT cells.

### 3.2. ATRT Cells Promote Exosome Released from tMSCs via a Paracrine Mechanism

**Figure 2:**
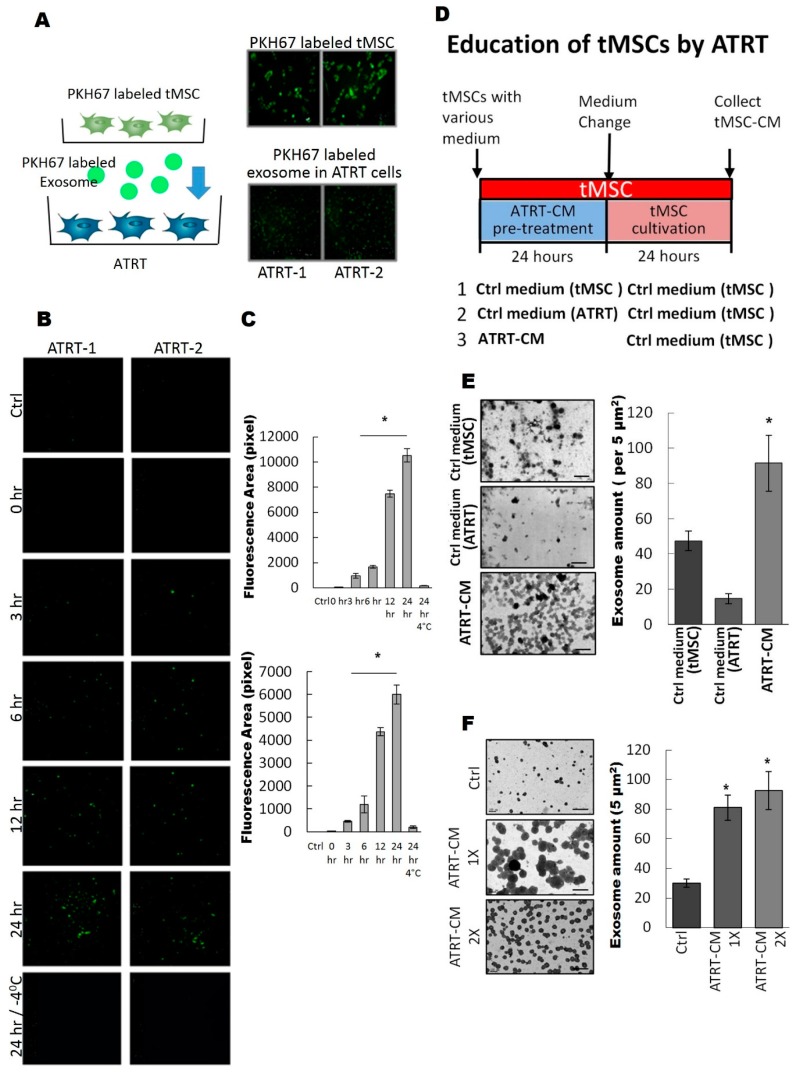
ATRT cells promote exosome released from tMSCs via a paracrine mechanism. (**A**) PKH67 green fluorescent-labeled tMSCs were indirectly co-cultured with ATRT-1 and ATRT-2 cells in the 0.2 µm filter trans-well system. Right: 24 h after the co-culture, the ATRT cells were observed under a fluorescence microscope to investigate the exosome uptake. Left: schematic presentation of the PKH67-labeling and indirect co-culture system. (**B**,**C**) PKH67 green fluorescent-labeled tMSCs-derived exosomes were cultured with ATRT-1 and ATRT-2 cells and cells were observed in a time-course manner for the exosome update of ATRT cells. ATRT cells co-cultured under −4 °C condition for 24 h served as a negative control. The fluorescent intensity was quantified by Image J and presented in the chart in (**C**). (**D**) Schematic illustration of the experimental design to investigate the effect on ATRT educated tMSCs. (**E**) Conditioned media from tMSCs pretreated with indicated media were collected and the exosome in each condition was visualized by TEM (left). Both the naïve culture medium for tMSC and ATRT cells serve as background controls. The number of exosomes released from tMSCs was quantified by Image J based on TEM photo and presented in the charts (right). This experiment was done with three distinct biological replicates. * *p* < 0.05. (**F**) tMSCs were pre-treated with low (CM-1X) and high (CM-2X) concentration of ATRT conditioned media for 24 h. Media were then replaced with tMSC culture medium for another 24 h. Naïve medium for ATRT culture was served as background control. Exosomes released from tMSC were observed under TEM and quantified by Image J based on TEM photo and presented in the charts (right).

We further examined whether tMSC-derived exosomes could enter ATRT cells. We firstly labeled tMSCs with green fluorescent lipid dye PKH67 on their lipid bi-layer membrane [28,29,30], so that the plasma membrane and all the extracellular vesicles containing lipid bi-layer membrane structure are all labeled with green fluorescence. In an indirect co-culture system with PKH67-labeled tMSCs and ATRT cells, we observed that after 72 h of co-culture, ATRT cells has taken up green fluorescent particles (Figure 2A). To further specify that it is exosome that transferred into ATRT cells, we labeled exosomes purified from tMSCs with PKH67 [28,29,30] and cultured these PKH67 labeled exosomes with ATRT cells in a time-course manner (Figure 2B,C). The uptake of PHK67-labeled exosomes in ATRT cells can be observed under fluorescence microscopy as early as three hours of culture, and reached maximal intake levels at 24 h, over 10 times higher than at three hours of incubation (Figure 2B,C). The low temperature condition inhibits the intake process of ATRT and, therefore, no fluorescence signal can be seen (Figure 2B,C). The overall PKH67 fluorescence intensity quantified by Image J (Figure 2C) indicate a time-dependent manner of the exosomal uptake.

Aggressive malignant cancer cells can inhabit the stroma where they can modify the metabolism of resident cells and transform stroma cells into tumor-associated stromal cells [34]. Therefore, unraveling the complex mechanisms of ATRT/tMSC paracrine crosstalk is important for understanding the tumor progression of ATRT. Hence, in this study, we aimed to investigate how ATRT cells transform and educate tMSCs to become cancer-associated stromal cells. The scheme of experimental design to evaluate the effects of ATRT on tMSCs is illustrated in Figure 2D. Briefly, ATRT cells cultured in control medium for either tMSC or ATRT were included as a control. After 24 h, all educated tMSCs under various treatments were washed and replaced with new tMSC medium. Finally, conditioned media were collected 24 h after being used as growing media for naïve ATRT cells (Figure 1D). After 24 h of the treatment, tMSC-CM-derived exosomes were harvested from various conditioned media including Ctrl medium (tMSC), Ctrl medium (ATRT) and ATRT-CM. Exosome amount was determined. The number of exosomes released from ATRT-educated tMSCs pretreated ATRT CM was significantly (two times) higher than of those from pretreated Ctrl media as quantified by Image J (Figure 2E). Especially, when tMSCs were pre-treated with one time (ATRT-CM-1X) or two times (ATRT-CM-2X) concentrations of ATRT conditioned media, the number of isolated exosomes was slightly increased in ATRT-CM-2X pretreatment (approximately a 10% increase) (Figure 2F), verifying that pretreatment of tMSCs with low concentration of ATRT conditioned medium is sufficient to educate tMSCs to secrete abundant exosomes.

### 3.3. Exosomal miR-155 Directly Targets SMARCA4 in ATRT Cells

**Figure 3:**
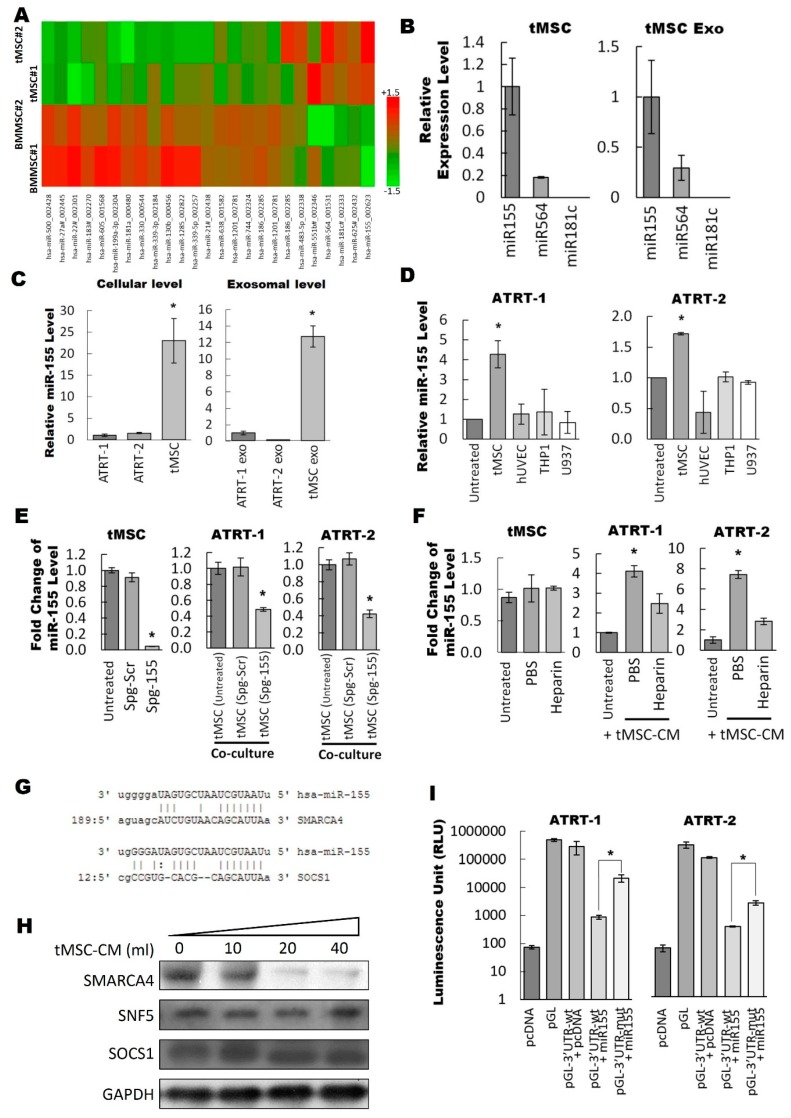
Exosomal miR155 suppressed the protein expression level of SMARCA4 in ATRT cells. (**A**) Exosomes in the conditioned medium of ATRT-educated tMSCs and bone marrow MSCs were collected by centrifugation. The total RNA was extracted and subjected to a miRNA microarray to detect the expressional changes of miRNA. (**B**) RNA extracted from tMSC (left) and tMSC-derived exosomes (right) were subjected to Taq-man quantitative real-time PCR analysis to evaluate the expression levels of indicated miRNAs. miR155 was the highest expressed within the three miRNAs. (**C**) The cellular (left) and exosomal (right) levels of miR155 in tMSC, ATRT-1, and ATRT-2 cells were detected by quantitative real-time PCR. Both cellular and exosomal levels of miR155 were higher in tMSC than in ATRT-1 and ATRT-2. (**D**) ATRT-1 (left) and ATRT-2 (right) were co-cultured with different types of stromal cells for 24 h. ATRT cells were harvested and total RNA was extracted for the evaluation of miR155 expression level by quantitative real-time PCR. The miR155 level in ATRT cells co-cultured with tMSCs was significantly higher than those co-cultured with other types of stromal cells. (**E**) MSCs were transfected with sponge miR155 (spg-155) or sponge scramble (spg-scr) before subjected to cellular miR155 level assessment by qRT-PCR (left). ATRT-1 (middle) and ATRT-2 (right) co-cultured with spg-scr or spg-155 transfected tMSCs were subjected to RT-PCR to analyze the cellular miR155 expression levels. (**F**) tMSC (left), ATRT-1 (middle) and ATRT-2 (right) were treated with either PBS or heparin for 24 h before subjected to qRT-PCR to analyzed cellular miR155 expression levels. (**G**) Search for miR155 targets by micro-RNA binding site database (microrna.org). *SMARCA4* and *SOCS1* were selected as strong candidates for exosomal miR155 targeting in ATRT cells. (**H**) ATRT-2 cultured in 40 mL medium contained a dose-course of tMSC conditioned medium (from 10 mL to 40 mL) were subjected to a Western blot analysis to evaluate the protein expression levels of miR155 targets, SMARCA4 and SOCS1, and ATRT biomarker, SNF5. Ctrl: 40 mL of fresh DMEM medium. (**I**) ATRT-1 (left) and ATRT-2 (right) transfected with wildtype or mutated *SMARCA4*- three prime untranslated region (3’UTR) reporter plasmids in the presence or absence of miR155 expression plasmids were subjected to luciferase assay.

Recently, it became increasingly clear that exosomes are able to shuttle genetic materials including mRNAs and miRNAs [12,14]. In this study, we concentrated on studying miRNAs as functional miRNAs (exo-miRNAs) transferred by exosomes to recipient cells are thought to be the main mediators of paracrine interaction between tMSCs and tumors [35]. To identify which exosomal miRNAs play important role in the interaction between ATRT and tMSCs, the ATRT educated-tMSC- and bone marrow MSC-derived exosomes were first isolated by ultra-centrifugation and extracted total RNA was subjected to a miRNA microarray profiling (Figure 3A). The criteria for the cut-off threshold in the microarray analysis was ≥1.5 or ≤−1.5. The miRNA microarray results showed that only seven out of 25 differentially expressed miRNAs (approximately 1/3 of differentially expressed miRNAs) were found to be expressed in the exosomes collected from ATRT educated tMSC-conditioned medium (Figure 3A). The expression of three highly upregulated miRNAs including miR-155, miR-564 and miR-181c was quantified in both tMSC exosomes and tMSC by using TaqMan real-time PCR analysis (Figure 3B). The results showed that miR-155 was the most highly expressed among the three miRNAs in both tMSC exosomes and tMSC cells. Therefore, miR-155 would be our main focus in the subsequent experiments. The relative miR-155 expression at the cellular and exosomal level of two ATRT cell lines and tMSC were first examined (Figure 3C). the endogenous level of miR-155 was abundant in tMSC and very low in both ATRT cell lines (Figure 3C, right panel). A similar observation was obtained at the exosomal level in which miR-155 was expressed 12 times higher in tMSC exosomes than in ATRT exosomes (Figure 3C, left panel). Next, to determine whether miR-155 expression in ATRT cells co-cultured with tMSCs was increased, the miR-155 expression was evaluated after 24 h of co-culturing ATRT cells with different types of stromal cells (Figure 3D). Among four tested stromal cells such as tMSC, ENDO, THP1 and U937, only co-culturing with tMSC lead to a significant elevation in miR-155 expression, implying that tMSC exosomes containing miR-155 cargo were released by tMSCs into the extracellular environment and then transferred into ATRT cells. We then confirmed our hypothesis either by blocking exosomal miR-155 through the introduction of sponge miR155 (Spg-155; Supplementary Figure S2) against miR-155 into MSCs (Figure 3E) or by treating with heparin to reduce exosome uptake by recipient cells (Figure 3F). Transfection of tMSCs with an spg-155 resulted in a significant reduction of the endogenous miR-155 level in tMSCs as compared with a sponge scramble antagomir (Spg-scr)-transfected control (Figure 3E, left panel). Furthermore, when ATRT-1 (Figure 3E, middle panel) or ATRT-2 (Figure 3E, right panel) were co-cultured with spg-155transfected MSCs, ~60% significant downregulation of expressed miR-155 in two ATRT cells was observed. Similarly to spg-155-mediated suppression of miR-155 expression in ATRT, co-culturing of ATRT-1 (Figure 3F, middle panel) or ATRT-2 (Figure 3F, right panel) in tMSC-derived conditioned medium in the presence of Heparin showed inhibition of the uptake of miR-155. As expected, the cellular expression of miR-155 in tMSCs was unchanged after treatment with heparin (Figure 3F, left panel). Taken together, the tMSC-derived exosome-carrying miR-155 cargo was able to enter ATRT cells.

We further investigated the functions of miR-155 in the regulation of tumor progression based on bioinformatics analysis to predict the miRNA-target mRNA interaction. We have employed a microRNA binding site database (microrna.org) to perform computational target analysis to mine for miR-155 potential target genes. Among 5445 predicted target genes of miR-155 (data not shown), *SMARCA4* and *SOCS1* have drawn our attention as strong candidates for exosomal miR-155 targeting in ATRT cells (Figure 3G). Tumor suppressor *SMARCA4* is usually silenced or mutated in tumors [36,37,38] and overexpression of *SMARCA4* in neuroblastoma cells diminishes cell viability [39]. *SOCS1*, on the other hand, is known to act as a tumor suppressor in various cancers as well as a potent inhibitor of inflammation [40]. Thus, the possible relationship between miR-155 and *SMARCA4* and/or *SOCS1* was further explored. Different amounts of tMSC conditioned medium from 10 mL to 40 mL were introduced to ATRT-1 culture. The protein levels of *SMARCA4* and *SOCS1* in ATRT-1 were assayed by Western blot (Figure 3H). As expected, ATRT biomarker, *SNF5* was expressed in all treatment conditions. When ATRT-1 cells were treated with various amounts of tMSC conditioned media, there were no obvious changes of SOCS1 protein levels but SMARCA4 protein level was slightly reduced and dramatically downregulated in 20 mL and 40 mL tMSC conditioned medium. (Figure 3H). Moreover, miR-155 was found to be highly expressed in tMSC exosomes (Figure 3A,B). Thus, our findings pointed out to an inverse correlation between miR-155 and SMARCA4. Luciferase assay was carried out to demonstrate whether SMARCA4 was a direct target of miR-155 (Figure 3I). The three prime untranslated region (3’UTR) of the SMARCA4 reporter plasmid encompassing putative miR-155-target site was co-transfected with the miR-155 mimic. The results showed a decrease to ~50% of luciferase activity compared to that in the control cells transfected with either mutated or blank vectors (Figure 3I). Taken together, our data verified that exosome-carrying miR-155 directly targeted SMARCA4 to downregulate its expression.

### 3.4. The Exosomal-miR155/SMARCA4 Pathway Regulates ATRT Migration Ability

**Figure 4:**
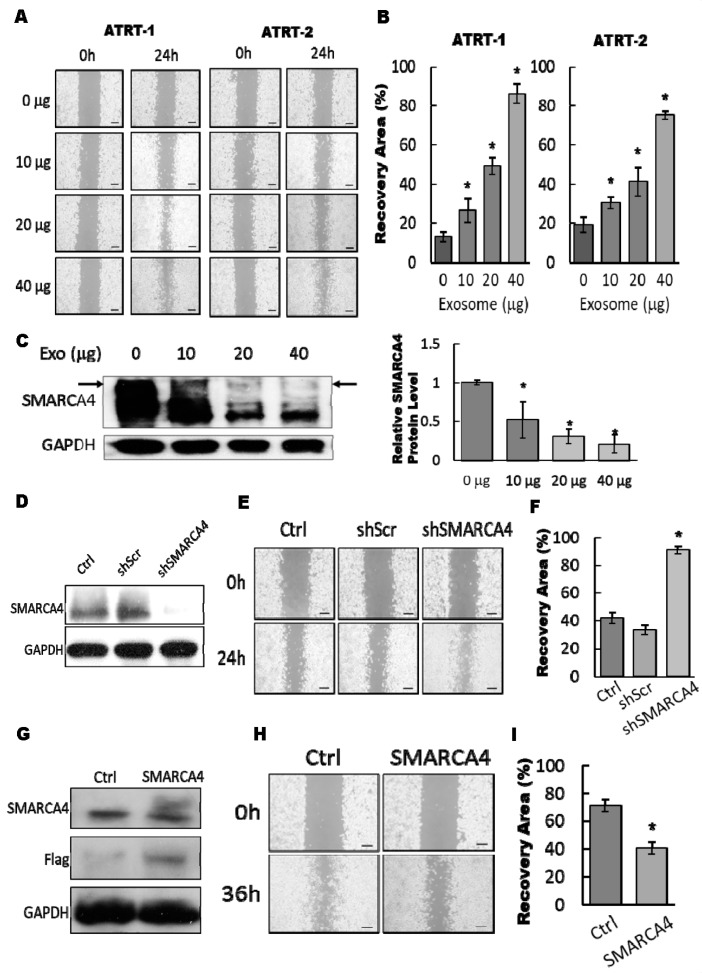
The exosomal-miR155/SMARCA4 pathway regulates ATRT migration ability. (**A**) ATRT-1 and ATRT-2 cells were subjected to a wound-healing migration assay in the presence or absence of a dose-course manner from 10 to 40 µg of purified tMSC-exosomes. The migrated cells were observed under a microscope for 24 h (A); the area covered by migrated cells were quantified by Image J and presented in the charts (B). This experiment was done with three distinct biological replicates. * *p* < 0.05. (**C**) ATRT-2 cells were treated by purified tMSC-exosome in a dose-course manner and then subjected to a Western blot to analyze SMARCA4 protein expression levels (left). The intensity of SMARCA4 blot was quantified and standardized with that of GAPDH and presented in the bar chart (right). (**D**) ATRT-2 cells were transfected with scrambled shRNA (Scr) or shRNA against SMARCA4 (shSMARCA4) and subjected to Western blot analysis to assess the knockdown efficiency of shSMARCA4. A non-transfected ATRT-2 served as a background control (Ctrl). (**E**,**F**) ATRT-2 cells transfected with shScr or shSMARCA4 were subjected to a wound-healing migration assay for 24 h (**E**). The area covered by migrated cells were calculated by Image J and presented in the chart (**F**). This experiment was done with three distinct biological replicates. * *p* < 0.05. (**G**) ATRT-2 cells were transfected with empty vector (Ctrl) or Flag-tagged SMARCA4 and subjected to Western blot analysis to assess the expression of exogenous SMARCA4. (**H**,**I**) ATRT-2 cells transfected with Ctrl or Flag-tagged SMARCA4 were subjected to a wound-healing migration assay for 36 h (**H**). The area covered by migrated cells were calculated by Image J and presented in the chart (**I**). This experiment was done with three distinct biological replicates. * *p* < 0.05.

Our findings demonstrated that ATRT-educated tMSC-derived conditioned medium improved the migratory ability of ATRT cell lines (Figure 1). Thus, we hypothesized that the exosomes from conditioned medium are the main regulators of ATRT migration capability. ATRT cells pretreated with different amounts (0 to 40 µg) of tMSC-derived exosomes were subjected to a wound-healing migration assay (Figure 4A,B). Treatment of tMSC-derived exosomes enhanced cell migration in a dose-dependent manner. The migration ability of ATRT cells was markedly increased up to 4-fold by pretreatment with 40 µg of exosomes in comparison to no exosomes control (Figure 4A,B). Furthermore, 80% downregulation of protein levels of SMARCA4 was observed when ATRT-2 cells were treated by 40 µg of purified tMSC-exosomes (Figure 4C). The findings indicated that tMSC-derived exosomes possibly mediated cell migration via SMARCA4 inhibition. The knockdown efficiency of SMARCA4 in ATRT-2 by shRNA targeting SMARCA4 (shSMARCA4) (Supplementary Table S3) was determined. Western blot analysis showed the efficient silencing of SMARCA4 by shSMARCA4 transfection (Figure 4D). Knockdown of SMARCA4 elevated ATRT-2 cell migration capability by ~80% after performing a wound-healing assay for 24 h (Figure 4E,F). In contrast, transfection of Flag-tagged SMARCA4 to overexpress SMARCA4 markedly uplifted the protein level in ATRT-2 (Figure 4G). When ATRT-2 cells transfected with Flag-tagged SMARCA4 were subjected to a wound-healing migration assay for 36 h, the ability of ATRT-2 to close the wound was diminished by ~20% (Figure 4I). In summary, our data indicated the exosome-mediated migratory ability of ATRT cells via attenuating SMARCA4.

### 3.5. Blocking of the Exosomal miR155/SMARCA4 Signaling Suppressed ATRT Migration and Tumor Growth

**Figure 5:**
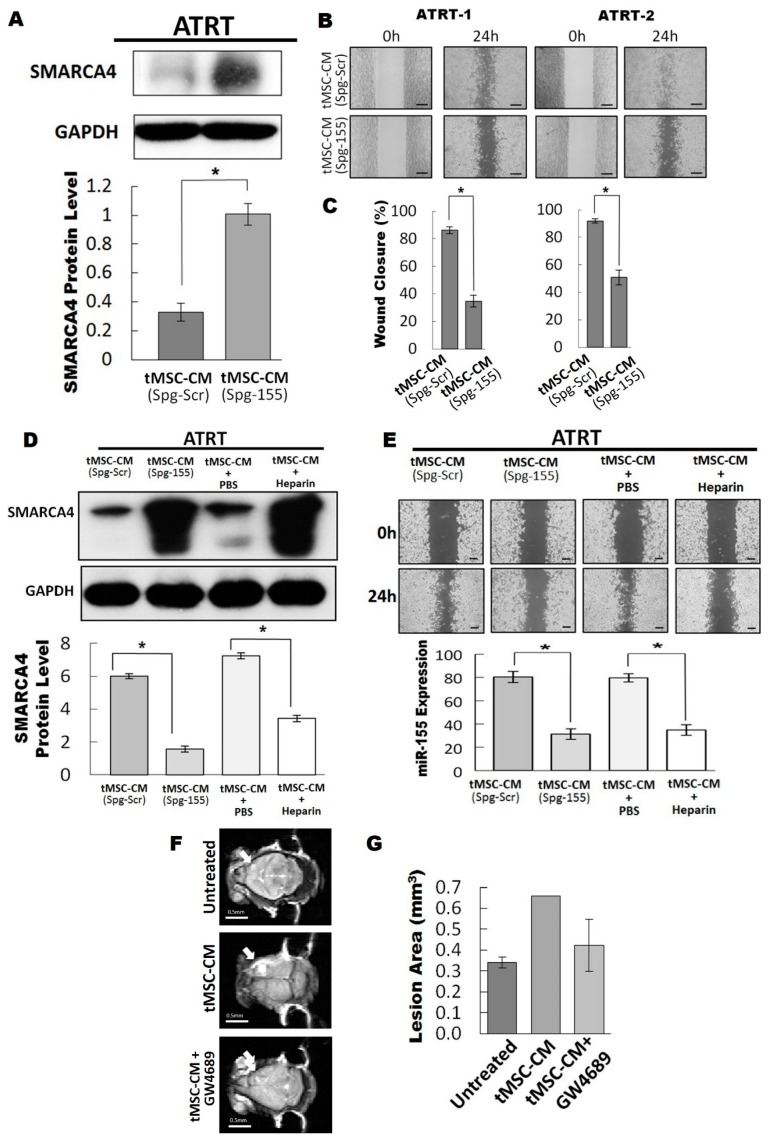
Blocking of the exosomal miR155/SMARCA4 signaling suppressed ATRT migration. (**A**) The tMSC was stably transfected with plasmids expressing scrambled or miR155 sponge (Spg-Scr and Spg-155, respective). ATRT-2 cells were treated with conditioned media collected from tMSC-transfected Spg-Scr and Spg-155 and subjected to a Western blot to assess the protein expression levels of SMARCA4 (top). The intensity of each blot was quantified by Image J and presented as relative levels in the chart (bottom). (**B–C**) ATRT-1 and ATRT-2 treated with conditioned media derived from tMSC which were transfected with plasmids expressing scrambled or miR155 sponge (SPG-Scr and SPG-155, respective) for 24 h were subjected to a wound-healing migration assay (B). The migrated cells covered area was calculated by Image J and presented as percentages relative to the initial area (C). (**D**) tMSC was transfected with either scrambled (Spg-Scr) or sponge miR155 (Spg-155), respectively. The tMSC was pre-incubated with Heparin or PBS for 2 h before the collection of the tMSC-CM. ATRT-2 cells were treated with tMSC-CM from various conditions for 24 h and analyzed by Western blot analysis to assess the protein levels of SMARCA4 (top). The intensity of each blot was quantified by presented as relative levels in the chart (bottom). (**E**) ATRT-2 cells with the same treatment as D were subjected to a wound-healing migration assay for 24 h (top), as well as a qRT-PCR for the expression levels of miR155 (bottom). (**F**) Immunocompromised mice were intracranially transplanted with ATRT-2 cells along with tMSC-CM in the presence or absence of GW4689 (10 μM). (**G**) Tumors were allowed to grow for 42 days and the tumor lesion area is measured by functional magnetic resonance imaging (fMRI).

Our results have shown that downregulation of *SMARCA4* is mediated by tMSC-derived exosomes carrying abundant miR-155. Furthermore, *SMARCA4* was shown to be a direct target of miR-155. Therefore, we hypothesized that tMSC-derived exosomes modulated ATRT migration via miR-155 transfer to silence *SMARCA4*. To test our hypothesis, we first transfected miR-155 sponge into tMSCs to inhibit the expression of miR-155 and subsequently, ATRT cells were co-cultured with CM collected from tMSC-silenced miR-155. *SMARCA4* was found to be highly upregulated as shown in western blot analysis (Figure 5A). Wound healing migration assay was then performed (Figure 5B) and the migratory ability of ATRT cells was quantified 24 h after co-culturing with tMSC-silenced miR-155-derived conditioned medium (Figure 5C). As expected, the capability of ATRT cells to migrate was slower than that of control samples when miR-155 expression in ATRT cells was diminished as a result of the introduction of spg-155, a miR-155 inhibitory plasmid, in tMSCs (Figure 5B). The migratory ability of ATRT cultured in spg-155-transfected tMSC-CM reduced wound closure by ~50% compared to those with the treatment of tMSC-transfected spg-scr-derived CM (Figure 5C). This might be explained by the fact that CM of tMSC-silenced miR-155 contained a low level of miR-155, therefore blockage of miR-155 transferred into ATRT cells cocultured with tMSC-transfected spg-155-CM promote endogenous *SMARCA4* expression resulting in slower wound healing rate. To support our assumption, the comparison between the expression of SMARCA4 and miR-155 upon co-culturing with tMSC-CM was made. Control treatments with PBS and Heparin were also included (Figure 5D). The results have shown the inverse correlation between SMARCA4 and miR-155 expression. When ATRT cells were treated with tMSC-CM, low expression SMARCA4 was in contrast to the high level of miR-155 and vice versa (Figure 5D). The findings obtained from wound-healing migration assays indicated that cells that underwent tMSC-CM treatments, including tMSC-CM (Spg-Scr) and tMSC-CM + PBS, migrated three times faster than those treated with CM but with either knockdown of miR-155 (tMSC-CM (Spg-155)) or after introducing Heparin (tMSC-CM + Heparin) (Figure 5E). Moreover, our in vivo experiments of 42-day intracranial injection of enriched tMSC-derived exosomes caused two times increased lesion area compared to the one injected with Ctrl or tMSC-derived exosomes with the addition of an exosomal inhibitor, GW4689 (Figure 5F).

**Figure 6:**
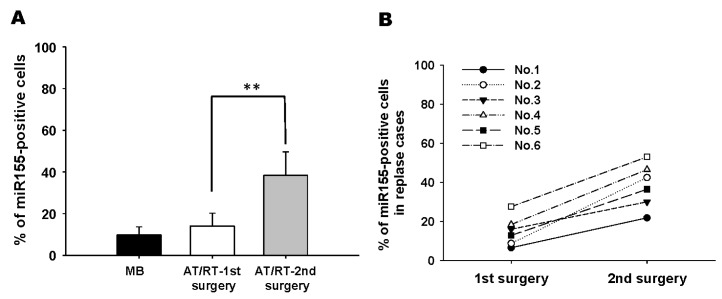
Correlation of miR155 levels and ATRT recurrence in clinical samples. (**A**) The percentage of miR155 + AT/RT cells (1st surgery: nine patients) was dramatically elevated in the tumor relapse samples (2nd surgery: six patients). (**B**) Comparison of the tumor samples from the first and second surgeries in the six patients whose tumors relapsed. ** *p* < 0.001.

Furthermore, we checked the expression levels of miR-155 in clinical samples from ATRT patients and found that the percentage of miR-155-positive ATRT cells was elevated in the samples from a second surgery in comparison to those from first surgery and medulloblastoma (Figure 6A). Similar results were also found in the pared ATRT samples from six patients who received a first and second surgery (Figure 6B). These data supported the fact that the high level of miR-155-positive ATRT cells is positively correlated with the occurrence of tumor relapse. Taken together, our results demonstrated that the migration of ATRT cancer and tumor growth were mediated by the uptake of tMSC-derived exosomes containing abundant miR-155, which in turn led to a downregulated expression of endogenous SMARCA4 in recipient cells.

## 4. Discussion

Carcinogenetic development and tumor progression are known to involve a series of oncogenic transformations that may be endowed by the tumor microenvironment. The tumor microenvironment is a complicated and heterogenetic niche that promotes cancer self-renewal and paracrine modulation for tumor recurrence, drug resistance, and metastasis [7,8]. The interactions between cancer stem-like cells and the tumor stromal cells can be very complex, which further dominated the tumor microenvironment to regulate the cancer metastasis [10]. Recent studies have discovered the crosstalk between cellular stemness pathways and tumor microenvironment, including extracellular matrix, micro-environmental cells, and extracellular stimuli. The tumor-associated stromal cells secrete stimuli, exosomes, and microRNAs to help cancer cells to evade immune responses and drug-induced cell death, whereas the paracrine factors and effects educate the normal stromal cells transforming them into tumor-associated stromal cells. Among the microenvironment components, tumor mesenchymal stromal cells have recently attracted remarkable interest due to their ability to migrate and engraft into areas of tumor development [41]. Indeed, mesenchymal stem cells were found to promote the formation of colorectal in the recipients [42,43]. In the present study, we demonstrated a tumor-environment-based paracrine regulation in which miR-155 suppressed SMARCA4 expression through enhancing tumor initiation. This report characterizes an exosomal miR-155-dependent crosstalk mechanism between tumor cells and stromal cells in the highly heterogeneous ATRT. Co-culturing the primary ATRT cells with AT-tMSC may target the former for malignant transformation and accelerate tumor growth. This crosstalk positively regulates the malignant properties of ATRT tumor cells and is responsible for the failure of current therapeutic treatment of ATRT patients.

The presence of exosomes in the microenvironment contributes to tMSCs—cancer crosstalk at long distance intercellular communication in a paracrine manner [13]. Exosomes transfer complex cargo containing proteins, lipids, specific mRNAs and regulatory miRNAs to neighboring cells [12,13,14]. After entering recipient cells, transferred exosomal mRNAs can be translated into proteins to modulate phenotypes of recipient cells, indicating to the natural function of exosomes as vehicles for genetic exchange between cells [44]. Interestingly, the study has also found that among 121 exosomal miRNAs from parental mast cells, some miRNAs were selectively and uniquely packaged into exosomes. Furthermore, recent studies have demonstrated the importance of the pathological crosstalk between tumor cells and their microenvironment via paracrine effects by secreting exosomes to promote tumor cell metastasis to reach distant organs [45,46,47,48]. Enforced expression of miR-105 in non-metastatic tumor cells resulted in enhancement of metastasis and induction of vascular permeability in distant organs [45]. Strikingly, metastasis in different organs seems to be site-specific as the expression of phosphatase and tensin homolog (PTEN) found in primary tumor cells is lost after spreading to the brain but not to other organs and recovered after evacuating brain microenvironment [49]. miRNAs from astrocytes were demonstrated to play the role of epigenetic regulators enforcing brain metastasis outgrowth [50]. The release of exosome-delivered miR-19a by astrocytes was shown to promote brain metastasis by elevating C-C Motif Chemokine Ligand 2 (CCL2) expression and recruiting myeloid cells via directly silencing PTEN expression. In vitro and in vivo lung cancer studies have revealed the contribution of miR-155 in a resistant phenotype by targeting Forkhead Box O3 (FOXO3A) and in an improvement of CSC properties [51]. Similar results were also obtained in breast cancer cells in which exosomal miR-155 mediated the chemoresistance phenotype [52]. Our current study expands knowledge of the involvement of MSC-derived exosomal miR-155 in contributing to tumorigenesis of ATRT through paracrine effects of MSC-ATRT crosstalk. Notably, in tMSC isolated from ATRT tissue, exosome-mediated transfer of tMSC-secreted miR155 efficiently increased self-renewal and tumor growth of ATRT through suppression of SMARCA4.

In liver cancer cells, transforming growth factor beta 1 (TGF-β1) indirectly promotes epithelial-mesenchymal transition (EMT) and CSC phenotypes through down-regulation of TP53INP1 by miR-155 [53] while in breast cancer, miR-155 is involved in tumor initiation via transcriptional repression of BRCA1 [54]. In this study, we demonstrated an in vitro tumor environment-based paracrine regulation in which MSC-derived exosomal miR-155 suppressed SMARCA4 expression through enhancing tumor initiation, and simultaneously miR-155 direct targeting brahma-related gene-1 (BRG-1) 3’UTR and secretion leading to the promotion of tumorigenicity. A potent regulatory role of miR-155 as tumorigenicity and proinflammatory inducer via mediating SMARCA4/BRG-1 expression was further supported by in vivo orthotopic model. These results suggested that exosome-miR-155-targeting axis may regulate the tumor metastasis and promotes progression in ATRT. Our findings are in agreement with other established works showing that miR-155 acts as an oncomiR to facilitate cell motility of human cancers by silencing tumor suppressor genes [53,54,55]. Our findings are supported by previous studies which described the stemness resistance mechanisms of miR-155. Given that abundant miR-155 expression in recurrent ATRT patients was detected and correlated with tumor progression, our study provided future research directions that may apply exosomal miR-155-based diagnostics and therapies for malignant ATRT.

## 5. Conclusions

**Figure 7:**
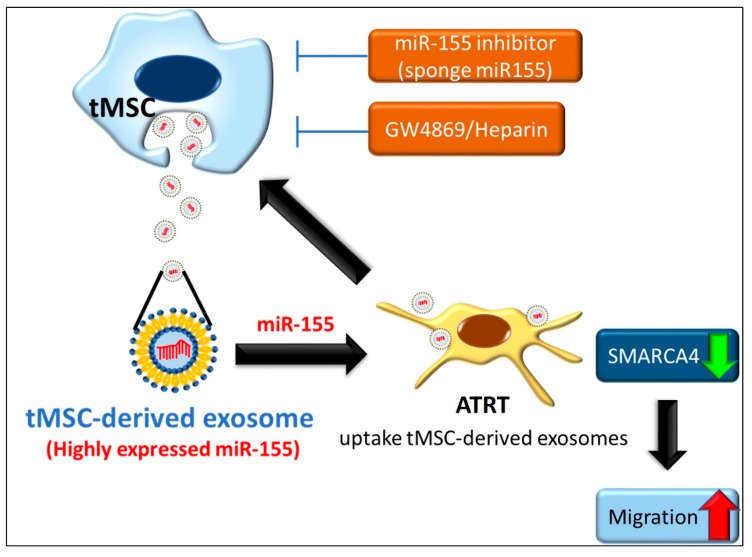
Schematic demonstration of paracrine interaction between MSCs and ATRT tumors. Briefly, tMSC-derived highly expressed miR-155- containing exosomes are transferred to ATRT cells. The abundant expression of exosomal miR-155 in ATRT leads to downregulation of SMARCA4, a direct target gene of miR-155. Hence, the migratory ability of ATRT increases. On the other hand, ATRT cells educate/stimulate tMSCs to release a higher amount of exosomes, and thus improve migration of ATRT cells. This malignant property of ATRT is reduced when miR-155 or exosome inhibitors are introduced into tMSCs.

The present study unveiled a novel paracrine interaction between tMSCs and ATRT tumors mediated by exosomal miR-155 (Figure 7). A tMSC-derived exosome contains a high level of miR-155 and is taken up by ATRT cells. The abundant expression of exosomal miR-155 is then transferred to ATRT cells and causes the downregulation of tumor suppressor gene SMARCA4, a direct target gene of miR-155, resulting in enhancement of the migratory ability of ATRT. On the other hand, ATRT cells also educate tMSC and stimulate tMSCs to release a higher amount of exosomes, and thus promote ATRT tumor growth in vivo and increase ATRT cell migration in vitro. The malignant properties of ATRT tumors are reduced when miR-155 sponge or exosome inhibitors, such as GW4869 and Heparin, are introduced to tMSCs. An exosomal miR-155-dependent crosstalk mechanism between tumor cells and stromal cells in the highly heterogeneous ATRT positively regulates the malignant properties of ATRT. Lastly, the identification of the exosome-based miR-155-mediated paracrine pathway in the present study provides a potential target for further development of successful therapeutic approaches for ATRT.

## Supplementary Materials

The following are available online at https://www.mdpi.com/2072-6694/11/5/720/s1, Figure S1: Characterization of tMSC surface markers, Figure S2: miR-155 sponge construction, Table S1: List of antibodies, Table S2: List of quantitative real-time PCR primers, Table S3: List of shRNA target sequences.
