# Supplementary material for: Tumor Mesenchymal Stromal Cells Regulate Cell Migration of Atypical Teratoid Rhabdoid Tumor through Exosome-Mediated miR155/SMARCA4 Pathway
Source: Cancers (Basel). 2019 May 24;11(5):720. doi: 10.3390/cancers11050720 (PMC6563126; doi:10.3390/cancers11050720)
Supplement: Supplementary file 1 [file cancers-11-00720-s001.pdf]

Supplemental Fig. 1

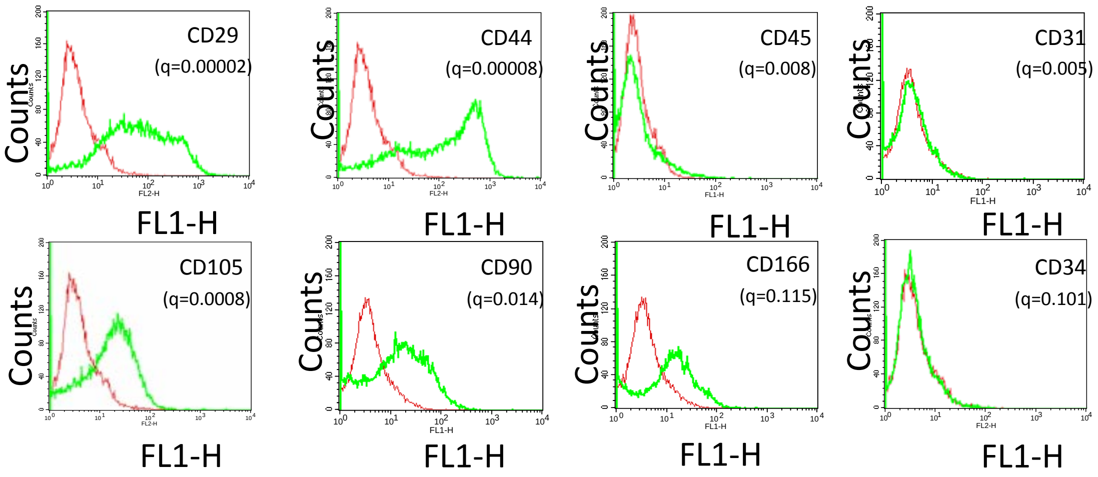

Suppl. Fig. 1. Characterization of tMSC surface markers.
The isolated tMSCs were subjected to flow cytometry analysis to assess the expression of surface markers. The isolated tMSCs expressed the MSC-specific markers (CD29, CD44, CD90, CD105, and CD166), but not the endothelial and hematopoietic cell markers (CD31, CD34, and CD45).

Supplemental Fig. 2

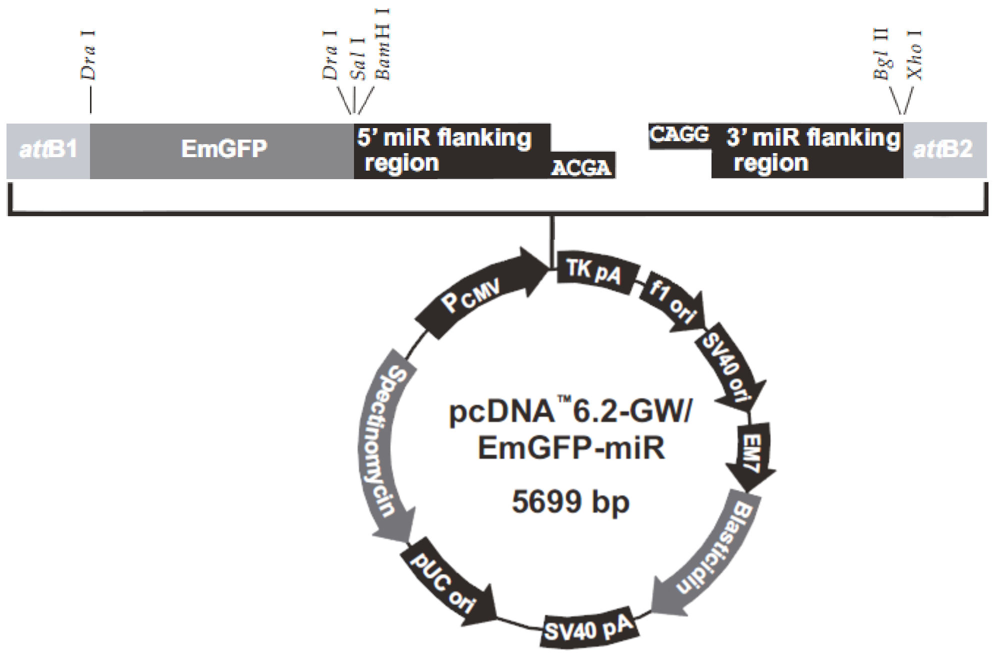

miR-Scramble-2XCtrl_T	**TGCTGTAACACGTCTATACGCCCAAATTTAACACGTCTATACGCCCAGTCC**
miR-155-B_2XSpg_T	**TGCTG**ACCCCTATGGAATTAGCATTAAAATTACCCCTATGGAATTAGCATTAAGTC C

Suppl. Fig. 2. mir-155 sponge construction.

Supplemental Table 1. List of antibodies.

Name	Host species	Company	Catalog NO.	Application
FLAG	Mouse mAb	Sigma	F3165	WB
SMARCA4	Rabbit mAb	Cell Signaling	#49360	WB
SNF5	Mouse mAb	Abcam	ab58209	WB
SOCS1	Rabbit mAb	Cell Signaling	#3950	WB
GAPDH	Rabbit mAb	Cell Signaling	#5174	WB

Supplemental Table 2. List of quantitative real-time PCR primers.

Name	Forward (5’ to 3’)	Reverse (5’ to 3’)	Tm (℃)
hsa-miR-155-5p	CGCAGTTAATGCTAATCGTGATAG	GGTCCAGTTTTTTTTTTTTTTTAACC	62.3
hsa-miR-181c-5p	GAACATTCAACCTGTCGGT	GGTCCAGTTTTTTTTTTTTTTTACTCA	57.6
hsa-miR-564	CAGAGGCACGGTGTCA	AGTTTTTTTTTTTTTTTGCCTGCT	57.8
SMARCA4	TACAAGGACAGCAGCAGTGG	TAGTACTCGGGCAGCTCCTT	60.0

Supplemental Table 3. List of shRNA target sequences

Name	Target sequence	Target gene
shSMARCA4	5'-GTGCGACATGTCTGCGCTG-3'	Human SMARCA4 gene
shScr	5'-CAGTTACTAGACGCGATCG-3'	Scrambled shRNA sequence as a control
